# Risk predictors of early recurrence in women with epithelial ovarian cancer in Lagos, Nigeria

**DOI:** 10.11604/pamj.2020.36.272.17827

**Published:** 2020-08-12

**Authors:** Kehinde Sharafadeen Okunade, Imoleayo Elijah Adetuyi, Muisi Adenekan, Ephraim Ohazurike, Rose Ihuoma Anorlu

**Affiliations:** 1 Department of Obstetrics and Gynaecology, College of Medicine, University of Lagos, Lagos, Nigeria,; 2 Department of Obstetrics and Gynaecology, Lagos University Teaching Hospital, Lagos, Nigeria

**Keywords:** Cytoreduction, epithelial ovarian cancer, FIGO stage, recurrence, Lagos

## Abstract

**Introduction:**

epithelial ovarian cancer (EOC) is the most lethal gynaecological cancer with a recurrence rate as high as 85% after an initial treatment. However, there are currently no reliable means of predicting the risk of recurrence after first-line treatment. This study investigated the risk factors that predict early recurrence of EOC after primary treatment among women in Lagos, Nigeria.

**Methods:**

this was a retrospective cohort study involving the review of all histologically confirmed EOC patients managed at the Lagos University Teaching Hospital, Lagos, Nigeria over a 7-year period from January 2010 to December 2016. A study proforma was used to retrieve relevant information and descriptive statistics were computed for all data. The associations between variables were tested and multivariate analysis was done to adjust for all the possible characteristics that predict early EOC recurrence.

**Results:**

the rate of recurrence of EOC was 76.4%. Suboptimal debulking surgery is the only independent predictor of early tumour recurrence.

**Conclusion:**

women should be adequately counselled and encouraged to report their symptoms early to ensure optimal primary treatment. Strategic efforts should also be made to further improve subspecialty training programs and skills development in gynaecological oncology in Nigeria and sub-Saharan Africa.

## Introduction

Ovarian cancer (OC) is the sixth most common cancer among women worldwide [1]. There are more than 200,000 new cases of OC diagnosed with approximately 6.6 new cases per 100,000 women per year [1]. Several studies in Nigeria and Africa [2-5] have shown that OC is the second most common gynecological cancer and it constituted about 7 to 8.2% of all gynecological malignancies [6, 7]. It is the second commonest cause of deaths among women admitted into the gynecological ward of a teaching hospital in Lagos [6]. Epithelial ovarian cancer (EOC) is the most lethal gynaecological cancer [8] with over 70% cases being diagnosed at an advanced stage [7, 9, 10]. The recurrence rate at this stage of the disease (International Federation of Gynecology and Obstetrics, FIGO stage III-IV) after an initial treatment and response is still as high as 85% [11]. However, there are currently no reliable means of predicting an individual´s risk of recurrence and its timing after first-line treatment for EOC and this limits physicians´ ability to provide appropriate risk-based surveillance follow-up [12]. On this basis, the current study was focused on investigating the clinicopathological and surgical risk factors that may predict early recurrence of EOC after primary treatment in black Nigerian women and then determine whether the presence or absence of the implicated factor(s) will play a role in the future management of these patients in our resource-limited settings.

## Methodss

This was a retrospective cohort study involving the review of case records of all histologically confirmed EOC patients managed at the Lagos University Teaching Hospital (LUTH), Lagos, Nigeria over a 7-year period from January 2010 to December 2016. LUTH is the largest tertiary institutions that provide services to patients in Lagos and from the neighbouring south-western states. The hospital is in the central Lagos metropolis and offers mainly clinical services among which include gynaecological oncology services. Study participants were women with histologically confirmed EOC. Eligibility criteria included women who had primary cytoreductive surgery and then adjuvant or adjunctive chemotherapy within 6-weeks of surgery. Patients with non-EOC; those who failed to complete primary treatment or yet to complete their 3 years follow-up were excluded from the study. The registration numbers of all women with EOC who were managed during the period under review were obtained from the gynaecological oncology ward registers and the patients´ case notes were subsequently retrieved from the medical records department. Relevant information such as sociodemographic characteristics, menopausal status, body mass index (BMI), presence of major co-morbidity (hypertension/diabetes mellitus/kidney disease), type of primary treatment (primary debulking surgery or neoadjuvant chemotherapy), extent of primary debulking (optimal or suboptimal), presence of significant ascites (≥ 500mls), FIGO stage of disease, histological subtype of tumour and tumour grade were extracted using a standardized proforma.

Tumour recurrence in this study was defined the presence of any of clinical, biochemical or radiological evidence of the disease within 36 months of completion of primary treatment. Evidence of the disease was defined clinically as presence of a pelvic tumour on bimanual pelvic examination; and/or biochemically as abnormal (>35IU/ml) or increasing serum CA-125 levels; and/or radiologically as the presence of a new pelvic tumour on abdominopelvic USS or CT scan. All relevant data were analyzed using SPSS version 23.0 statistical package for windows manufactured by IBM Corp., Armonk, NY, United States. Descriptive statistics were then computed for all baseline patients´ characteristics. Characteristics of patients were described by mean and SD (if normally distributed) or median and percentiles (if distribution is skewed) for continuous variables and by frequencies and percentages for categorical variables. Associations between continuous variables were tested using the independent sample t-test (normal distribution) or the Mann-Whitney U test (skewed data), whereas categorical variables were compared using the chi-square (χ2) test or the Fisher exact test, as appropriate. Multivariate analysis was conducted with variables that had bivariate P-value < 0.2, to adjust for confounders in the relationship between the various risk factors and early recurrence of EOC. All testing was two-sided, and a P-value of less than 0.05 was considered to indicate statistical significance.

**Ethical approval:** ethical approval for the study was obtained from the Health Research and Ethics Committee of the Lagos University Teaching Hospital (ADM/DCST/HREC/1912) prior to the review of case records and data collection. Ethical principles according to the Helsinki declaration were considered during the study. The investigators ensured strict confidentiality of all patients’ information.

## Results

We recorded 81 cases of EOC managed in the hospital during the period under review with 72 cases being eligible or had their complete clinical data available for final analyses. Excluded from the final analysis were 5 women who failed to undergo complete primary treatment and 4 women who were lost to follow-up. The mean age of women in the study was 54.6 ± 10.7 years with the majority (66.7%) of them aged 50 years and above. Almost three-quarter (73.6%) of the women presented with advanced tumour and the commonest histological type of the disease was serous EOC (66.7%). Fifty-five (76.4%) of the women had tumour recurrence during the period under review with a median recurrence time of 15.0 (IQR 8.0, 29.5) months (Table 1). In Table 2, a subgroup analysis of the study cohorts who had tumour recurrence during the period of review, over one-half (54.5%) of the recurrence occurred within 12-months of treatment. Presence of medical co-morbidity (P = 0.012), suboptimal surgical cytoreduction (P = 0.028) and high tumour grade (P = 0.032) were positively associated with higher risks of early tumour recurrence within 12-months of primary treatment. However, after controlling for age, parity, co-morbidity, initial serum CA125, type of primary treatment offered and tumour grade, suboptimal surgical cytoreduction was found to be the only independent predictor of early tumour recurrence (RR-8.4, 95% CI-1.44, 48.87; P = 0.018) (Table 3).

**Table 1:** baseline characteristics of study cohorts (n = 72)

Variable	Frequency	%
**Age, in years**		
<50	24	33.3
≥50	48	66.7
**Mean age ± SD = 54.6 ± 10.7 years**		
Menopausal status		
Premenopausal	33	45.8
Postmenopausal	39	54.2
**Parity**		
Low parity (≤1)	23	31.9
High parity (≥2)	49	68.1
**Median parity (IQR) = 2.0 (1.0, 4.0)**		
Body Mass Index (BMI), in kg/m2		
≤25.0	37	51.4
>25.0	35	48.6
**Median BMI (IQR) = 24.7 (22.1, 28.9) kg/m2**		
Co-morbidity		
No	55	76.4
Yes	17	23.6
**Pre-treatment CA125 levels, in U/Ml**		
<250.0	25	34.7
≥250.0	47	65.3
**Median CA125 (IQR) = 437.0 (144.0, 924.5) U/mL**		
Type of cytoreductive surgery		
Optimal	37	51.4
Suboptimal	35	48.6
**Presence of significant ascites**		
No	37	51.4
Yes	35	48.6
**FIGO stage**		
Early (I-II)	19	26.4
Advanced (III-IV)	53	73.6
**Tumour grade**		
Low grade (grade I)	20	27.8
High grade (grade II-III)	52	72.2
**Histological type**		
Serous	48	66.7
Non-serous	24	33.3
**Type of primary treatment**		
Primary debulking	39	54.2
Neoadjuvant chemotherapy	33	45.8
**Recurrence within 36-months**		
Yes	55	76.4
No	17	23.6
Median PFS (IQR) = 15.0 (8.0, 29.5) months		

Abbreviations: SD, standard deviation; IQR, interquartile range; FIGO, international federation of obstetrics and gynaecology

**Table 2:** bivariate analysis of patients’ characteristics and timing of tumour recurrence (n = 55)

	Recurrence		
Characteristics	Early (%)	Late (%)	P-value
**Age, in years**			**0.108a**
<50	7 (23.3)	9 (36.0)	
≥50	23 (76.7)	16 (64.0)	
Mean age ± SD	56.9 ± 10.2	51.9 ± 12.4	
**Menopausal status**			**0.843**
Premenopause	14 (46.7)	11 (44.0)	
Postmenopause	16 (53.3)	14 (56.0)	
**Parity**			**0.600b**
Low	7 (23.3)	12 (48.0)	
High	23 (76.7)	13 (52.0)	
Median parity (IQR)	2.0 (1.8, 3.0)	2.0 (0.0, 4.0)	
**Body Mass Index (BMI), in kg/m^2^**			**0.846b**
≤25.0	14 (46.7)	12 (48.0)	
>25.0	16 (53.3)	13 (52.0)	
Median BMI (IQR)	25.2 (23.6, 29.7)	26.1 (21.0, 33.2)	
**Co-morbidity**			**0.012**
No	25 (83.3)	13 (52.0)	
Yes	5 (16.7)	12 (48.0)	
**Pre-treatment CA125 levels, in U/mL**			**0.147b**
<250.0	7 (23.3)	5 (20.0)	
≥250.0	23 (76.7)	20 (80.0)	
Median CA125 (IQR)	443.1 (339.0, 1110.0)	543.0 (449.1, 1191.5)	
**Type of primary treatment**			**0.101**
Primary debulking	8 (26.7)	12 (48.0)	
Neoadjuvant chemotherapy	22 (73.3)	13 (52.0)	
**Extent of cytoreductive surgery**			**0.028**
Optimal	7 (23.3)	13 (52.0)	
Suboptimal	23 (76.7)	12 (48.0)	
**Ascites**			**0.803**
No	13 (43.3)	10 (40.0)	
Yes	17 (56.7)	15 (60.0)	
**FIGO stage**			**0.202c**
Early	0 (0.0)	2 (8.0)	
Advanced	30 (100.0)	23 (92.0)	
**Disease grade**			**0.032**
Low grade	5 (16.7)	0 (0.0)	
High grade	25 (83.3)	25 (100.0)	
**Histological type**			**0.309**
Serous	24 (80.0)	17 (68.0)	
Non-serous	6 (20.0)	8 (32.0)	
Total	30 (54.5)	25 (45.5)	

Abbreviations: SD, standard deviation; IQR, interquartile range; BMI, body mass index; FIGO, international federation of obstetrics and gynaecology a Independent sample T-test; b Mann-Whitney U test; c Fisher’s exact test

**Table 3:** multivariate analysis of the factors associated with early recurrence

Characteristics	Early recurrence (≤12 months)		P-value
	Relative Risk (RR)	95% CI	
Age	0.98	0.93 - 1.05	0.608
Parity	0.90	0.62 - 1.31	0.580
Co-morbidity	0.44	0.07 - 2.87	0.387
Pre-treatment CA125 levels	1.00	0.99 - 1.00	0.858
Cytoreduction	8.37	1.44 - 48.87	0.018
Primary treatment	0.97	0.21 - 4.53	0.968
Tumour grade	N/A	N/A	0.999

Abbreviations: CI, confidence interval; N/A, not available

## Discussion

The rate of recurrence of EOC in the cohorts of women reviewed for this study was 76.4% with over half (54.5%) developing the recurrence within 12-months of treatment. The study found over 8-fold risk of early tumour recurrence in women who had suboptimal surgical cytoreduction. The recurrence rate of 76.4% in this study is only slightly higher than the range of figures (70-75%) reported in other previous studies conducted outside of Africa [13, 14] but significantly much higher than the 53.4% recorded in a 5-year retrospective review conducted by Amate *et al*. [15] in Paris, France. The variation in our study and others may be attributed to the higher rate of advanced disease seen mostly in our women who usually do not report their symptoms early as observed in the study. Moreover, the high rate of recurrence seen in most studies is still a pointer to the extreme difficulties encountered in tackling this disease which has so far not had any reliable screening test that will aid its early detection in low-risk group of women. The mean age of 54.32 ± 11.07 years recorded in the current study was similar to the median age of 54 (25-84) years reported by Liu *et al*. [16] in a prognostic study of EOC but quite higher than the mean of 45.7 ± 4.3 years we reported in a previous study carried out in the same setting in Lagos [7]. This difference is likely due to the restriction of the women in the present study to only those with EOC who are usually of a higher age group compared to those with other histological types of ovarian cancers such as germ cells or sex cord-stromal tumours. However, similarly to the work of Gil-Ibáñez *et al*. [17], age was not a significant predictor of ovarian cancer relapse in our study and this is a sharp contrast to the finding in a Taiwanese study by Chang and colleagues [18].

According to the gynecologic oncology group (GOG), optimal debulking surgery is defined as when the residual tumour implants are less than 1cm [19]. Previous studies have shown that the ability to achieve optimal debulking is the most important prognostic factor for ovarian cancer recurrence and survival [19-21] and this was also corroborated by our current study. We found in our study that early recurrence of EOC was significantly associated with the extent of surgical cytoreduction and this was corroborated by Spiliotis *et al*. in Greece [22] but unlike the finding reported by Paik *et al*. [23] who did not show any association. We, however, did not record a higher risk of tumor recurrence in women with advanced FIGO stage of the disease in similarity to the finding by Gil-Ibáñez *et al*. [17] but at variance with report from another study by Yan *et al*. in 2005 [24] where FIGO stage has been noted to be an independent risk factor of EOC recurrence. The major limitation of this study was its retrospective design that depended on effective documentation of patients´ history. The poor record keeping practice in our setting resulted in the unacceptably high number of incomplete clinical data and thus, the small number of cases eventually reviewed may not allow for the detection of very small effect. However, this is a pilot study that is meant to generate preliminary data for a future robust, multicenter and long-term longitudinal study to investigate these risk factors among women with ovarian cancer in Nigeria and Africa.

## Conclusion

This study reported a very high rate of recurrence among Nigerian women with EOC. It further revealed that suboptimal cytoreduction is the only independent predictor of early EOC recurrence in Nigerian women. We, therefore, suggest that women should be adequately counseled and encouraged to report symptoms early to ensure optimal primary treatment. Strategic efforts should also be made to further improve subspecialty training programs and skills development in gynecological oncology in Nigeria and sub-Saharan Africa.

### What is known about this topic

- Epithelial ovarian cancer (EOC) is the most lethal gynaecological cancer with a recurrence rate as high as 85% after an initial treatment;
- There are currently no reliable means of predicting the risk of recurrence after first-line treatment.

### What this study adds

- We reported a very high rate of recurrence among Nigerian women with EOC;
- A high serum CA125, suboptimal cytoreduction and an advanced FIGO stage were predictors of EOC recurrence;
- The development of a prognostic-algorithm for recurrence that comprises of the important predictors identified in this study may have a value in the follow-up of women with EOC after primary treatment.
